# High deforestation trajectories in Cambodia slowly transformed through economic land concession restrictions and strategic execution of REDD+ protected areas

**DOI:** 10.1038/s41598-022-19660-0

**Published:** 2022-10-14

**Authors:** Maren Pauly, Will Crosse, Joshua Tosteson

**Affiliations:** Department of Project Impacts, Everland LLC, New York, USA

**Keywords:** Climate-change impacts, Climate-change mitigation, Conservation biology, Climate-change policy

## Abstract

Protected Areas (PAs) are continuously being established in tropical forests in an effort to preserve biodiversity and reduce deforestation. It was recently demonstrated that PAs are more effective at reducing forest loss than unprotected control sites across southeast Asia. The voluntary REDD+ scheme offers a new framework for the protection of high deforestation landscapes, jurisdictions, and countries backed by international carbon finance. Here we analyzed the economic drivers of deforestation in Cambodia and the effectiveness of 3 REDD+ projects vs. adjacent protected areas. We find that Economic Land Concessions were a predominant driver of deforestation in Cambodia and influenced the trajectory of illegal forest conversion in PAs. Furthermore, REDD+ projects offer significantly more protection against deforestation than adjacent PAs in two of the three analyzed cases, likely due to enhanced funding enabling implementation of targeted community activities and rigorous monitoring and enforcement.

Despite global agreements to reduce deforestation, forest loss is progressing across the tropics at an alarming rate^1^. This is particularly concerning in light of the imperative to halt deforestation completely within this decade in order to achieve the Paris Agreement goal of stabilizing global warming at 1.5°C^2^.

In an attempt to reduce deforestation, terrestrial protected areas are being established to conserve the final remaining large expanses of tropical forests, which are currently releasing significant amounts of carbon (1.1PgC/yr) as a result of land use change^3^. In theory, protecting vast areas of tropical forest would be a credible solution—safeguarding both carbon and biodiversity. However, protection on paper does not necessarily translate to protection on the ground—particularly in regions of limited resources, high background deforestation and low rule of law. Unsustainable land use change tends to be more profitable than leaving the forest standing^4^—making it difficult to effectively prevent forest loss through policy interventions alone. The question therefore remains—how effective are national protected areas in reality?

In response to this, a recent paper by Graham et al. (2021)^5^ was published entitled, “Southeast Asian protected areas are effective in conserving forest cover and forest carbon stocks compared to unprotected areas”. The authors analyzed deforestation and resulting carbon emissions in protected areas^6^ compared to adjacent non-protected control sites using Hansen tree cover loss data^7^ (2000–2018). They demonstrated that the protected areas studied avoided significantly more forest loss than adjacent non-protected control areas. Malaysian and Cambodian protected areas avoided 15% and 11% of forest loss, respectively, compared to a matched counterfactual; effectively reducing more deforestation than the other countries analyzed, including Vietnam, Laos, Thailand, Indonesia and Myanmar.

Voluntary market-based REDD+ (Reduce Emissions from Deforestation and forest Degradation) provides a complementary avenue for forest protection by providing performance-based financial incentives for projects and jurisdictions to reduce forest loss against a defined baseline. In many cases, REDD+ is applied to protected areas under substantial deforestation risk. REDD+ has the potential to preserve natural forests by using carbon finance to transform the economic relationship between people, the governments and forests on the ground by making the forest more valuable in its standing state. In order to achieve this transformation, REDD+ projects develop activities and incentive structures through benefit sharing and the development of alternative sustainable livelihood opportunities (e.g. intensified sustainable agriculture, eco-tourism) whilst enacting robust protection measures^8^. It involves a rigorous monitoring, evaluation, and independent verification process, allowing REDD+ projects to access financial resources through the voluntary carbon market based on their level of performance.

Here, we explore the effectiveness of Cambodian REDD+ project areas in reducing deforestation compared to their adjacent non-REDD+ protected areas, whilst investigating the economic and political drivers of deforestation. In Cambodia, the number of protected areas (PAs: e.g. national parks and wildlife sanctuaries: IUCN Management Category IV) have been expanded across the country in an effort to safeguard the country’s remaining biodiverse forests^9,10^, many of which are Key Biodiversity Areas (KBAs: Supplementary Fig. 1)—serving as important wildlife habitats and corridors^11^. However, these PAs are chronically under-funded, limiting on-the-ground presence needed to effectively control deforestation and wildlife poaching^12,13^. We hypothesize that when applied to threatened forests in Cambodia, REDD+ can provide the financial support and capacity for PAs and other landscapes to reduce deforestation by using the financial proceeds to strengthen delivery of conservation outcomes in lieu of forest conversion and degradation.

Three REDD+ projects are currently registered in Cambodia—the Keo Seima Wildlife Sanctuary REDD+ Project (KSWS), the Tumring REDD+ Project (TRP) and the Southern Cardamom REDD + Project (SCRP) (Fig. 1a, Supplementary Table 1, Supplementary Fig. 2). The KSWS REDD+ project was developed in 2010 within the core area of what was the Seima Protection Forest, later becoming the Keo Seima Wildlife Sanctuary (Protected Category IV), covering parts of Mondulkiri and Kratie Provinces. This was followed by the TRP in 2015 (encompassing a series of Community Forests: Protected Category VI, and non-protected land) along the edge of Prey Lang Wildlife Sanctuary and the SCRP in 2015 covering sections of Southern Cardamom National Park (Protected Category II) and Tatai Wildlife Sanctuary (Protected Category IV) within the Cardamom Mountains rainforest. All project regions are under substantial threat of deforestation due to unsustainable extraction of wood and wildlife, continued expansion of the agricultural frontier, economic land concessions and illegal land grabbing enabled by local corruption.

**Figure. 1:**
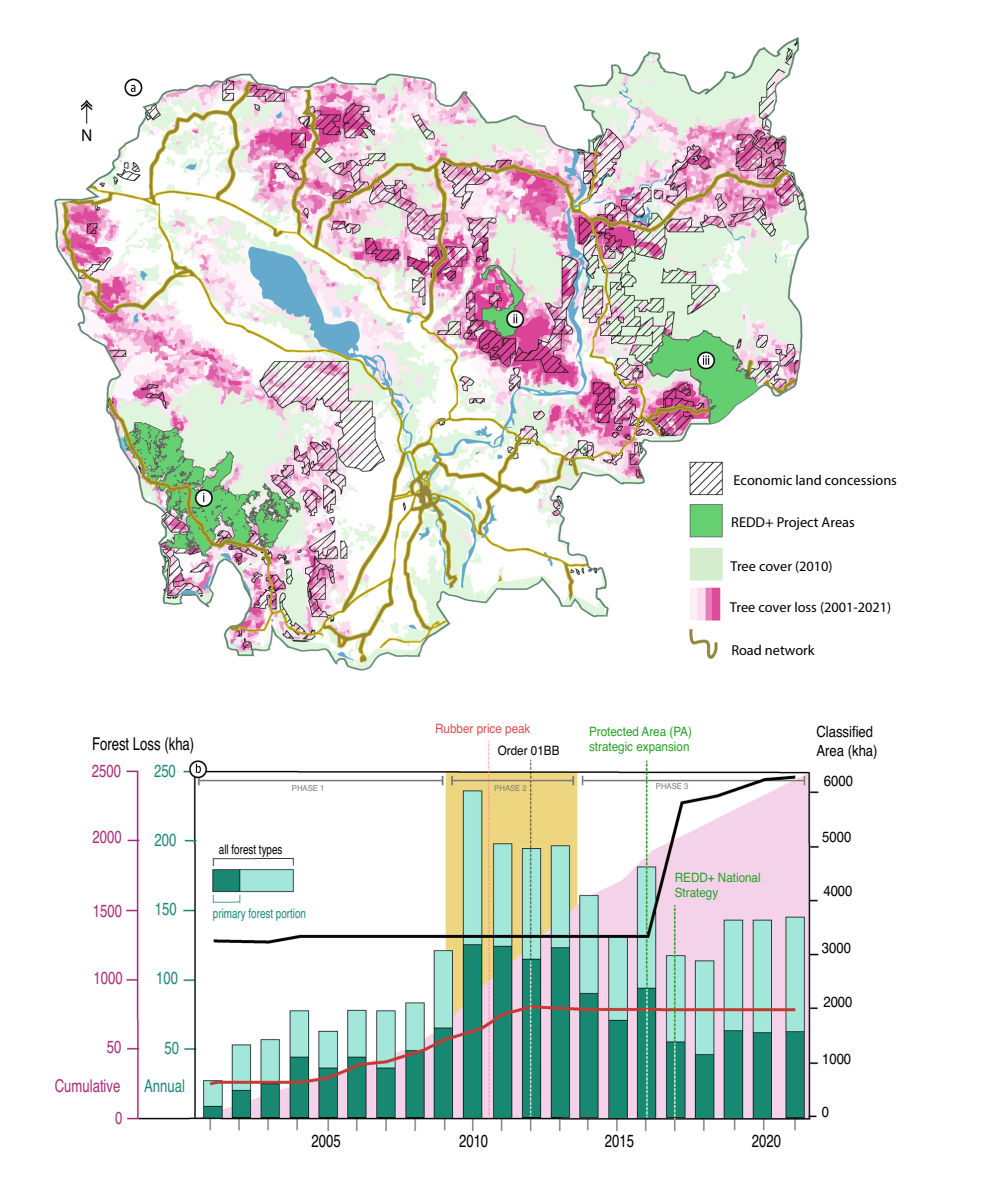
Deforestation drivers in Cambodia. (**a**) Tree cover loss (2001–2021^7^) with dark areas representing deforestation hotspots, CCB REDD+ project locations (i: Southern Cardamom, ii: Tumring, iii: Keo Seima) and Cambodia land concessions^25^. (**b**) Annual (bars) and cumulative (shaded area) deforestation in Cambodia from 2001 to 2021^7^, occurring in 3 phases with peak deforestation from 2010 to 2013 correlated to a spike in rubber price^14^ and peak in hectares of land classified as Economic Land Concessions (ELCs; red line)^25^. Hectares classified as Protected Area in black^6^. Basemap and layers accessed from Global Forest Watch (Creative Commons CC BY 4.0)^15^, finalized on Adobe Illustrator (v26.2.1)^16^.

## Results

### Country-level deforestation

Cambodia underwent significant deforestation in the past two decades, equal to 2.6 million hectares of forest cover loss since 2001 (Fig. 1b). Forest loss was higher in the most recent decade (2011–2021: 155,000 ha/year, 1.76%/year, total: 1.7 million ha) compared to the previous (2001–2010: 88,000 ha/year, 1.00%/year, total: 885,000 ha). Cambodia demonstrated the highest percentage of primary forest loss in 2021 (1.5%) on a country-level.

Forest loss in Cambodia occurred within three main phases, demonstrated using changepoint analysis—starting with a steady increase from 2000 to 2009 (Phase 1: + 0.81% annual increase over 9 years) before a significant increase occurred between 2009–2010 (+ 1.3% in 1 year) resulting in a series of peak deforestation years from 2010 to 2013 (Phase 2: 2.3%/year average) aligned with a 2010–2011 peak in rubber price (Fig. 1b). Following these peak years, deforestation rates moderately decreased (−31%), averaging 1.6%/year from 2014 onward (Phase 3); still twofold higher than the pre-peak (2000–2009) levels (0.82%/year). In total, Cambodia lost 29.5% of forest cover within the study period (2001-2021).

These three deforestation phases are aligned with trends of Economic Land Concession (ELC) activity in Cambodia. During Phase 1, ELCs increased by an average of 79,196 ha/year (2000–2009); in Phase 2, ELCs increased by an average of 130,000 ha/year; and by Phase 3, ELCs decreased by an average of nearly −67,000 ha/year. Correlation analysis has revealed a highly significant positive relationship between the hectares of ELCs and deforestation in Cambodia (R = 0.87, *p* < 0.0001, 2000–2021).

In recent decades, Protected Areas (PAs) have been established across Cambodia (Fig. 1b), with a particularly significant increase in PA establishment from 2016 onward (2010: ~ 3.3 million ha, 2016: ~ 5.9 million ha, 2021: ~ 6.3 million ha). A significant negative relationship was observed between the hectares of protected area forest and country-level deforestation (R = −0.62, *p* < 0.05, 2010–2021).

### Project-level protection

**Figure 2:**
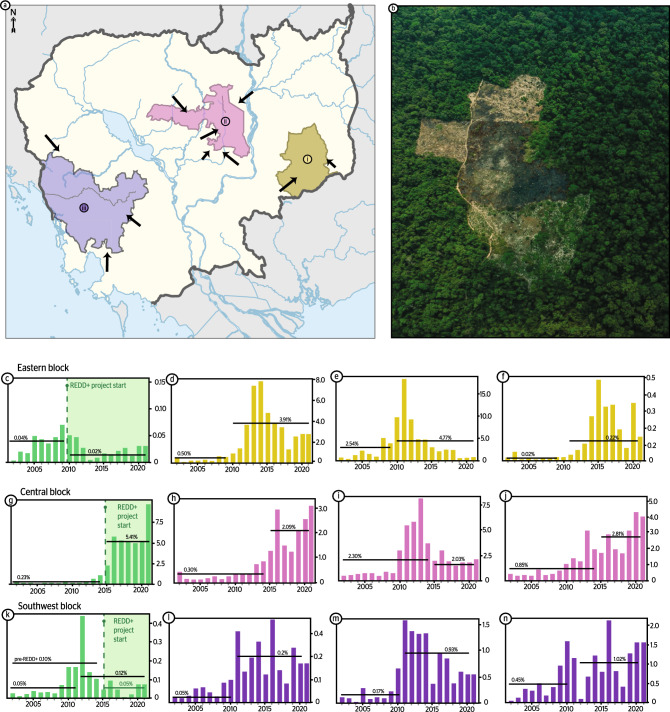
A snapshot of deforestation in three protected area blocks in Cambodia. 12 protected areas and REDD+ projects were analyzed in (**a**) Cambodia in three protected area blocks: (i) eastern, (ii) central, and (iii) southwest; arrows highlight deforestation frontiers. (**b**) An example of deforestation within the central block (credit: Filip Agoo on behalf of Everland LLC). Annual forest loss (% of total forest cover, 2001–2021^7^) was analyzed for: (**c**) Keo Seima REDD+ Project, (**d**) Keo Seima Wildlife Sanctuary, excluding the core forest, (**e**) Snuol Wildlife Sanctuary, (**f**) Phnom Prech Wildlife Sanctuary), (**g**) Tumring REDD+ Project, (**h**) Prey Lang Wildlife Sanctuary, (**i**) Beng Per Wildlife Sanctuary, (**j**) NorthWest Biodiversity Corridor Segments NWC2, NWC3), (**k**) Southern Cardamom REDD+ Project, (**l**) Phnom Kravanh National Park, (**m**) Phnom Aural Wildlife Sanctuary, and (**n**) Phnom Samkos Wildlife Sanctuary. See Supplementary Fig. 2 for relative locations. Basemap and layers accessed from Global Forest Watch (Creative Commons CC BY 4.0)^15^, finalized on Adobe Illustrator (v26.2.1)^16^.

Three protection blocks were analyzed—the eastern, central and southwestern blocks (Fig. 2, Supplementary Fig. 2, Supplementary Table 2)—housing the Keo Seima REDD+ project, the Tumring REDD+ project and the Southern Cardamom REDD+ project, respectively.

#### Eastern block

The eastern protected area block region (block+ 10 km buffer) has lost a total of 15.4% forest cover in the past two decades (2001–2021), representing a 48% lower rate than the country-level average. The average annual forest loss rate has nearly doubled from 0.45 from 2001 to 2010 to 0.99% from 2011 to 2021. Within the eastern protected area block (Fig. 2ai), a considerable deforestation frontier is evident—extending from the south at Snuol Wildlife Sanctuary (SWS: Protected Category IV) through the borders of Keo Seima Wildlife Sanctuary (excluding the core forest = KSWS; Protected Category IV) and eventually into the Keo Seima REDD + core zone (KSRP). This is demonstrated in the first decade with the highest deforestation rate in Snuol (2001–2010 average = 2.54%) followed by KSWS (2001–2010 average = 0.50%) and eventually the KSRP (2001–2010 average = 0.04%). Deforestation rates continued to rise in the aforementioned two protected areas (+ 188% and + 781%, respectively) during the recent decade—leading to a de-gazetting of SWS—resulting in a deforestation hotspot (2010–2021: KSWS + SWS average annual = 4.56%, Cambodia average = 1.84%). At the same time, deforestation declined in the KSRP (−50%) in association with the initiation of the project in 2010. Within the study period, SWS lost 73.1% forest cover, followed by 18.9% at KSWS and 0.61% within the KSRP. A significant positive correlation was observed between forest loss at SWS and KSWS with a 3-year delay (R = 0.82, *p* < 0.0001), signifying the movement of the deforestation frontier. No significant correlation was found between the KSRP and SWS.

Since 2016, another deforestation hotspot started to develop near the eastern borders of KSWS, close to the adjacent Phnom Prich Wildlife Sanctuary (PPWS: Protected Category IV) and a segment of the Northwest Biodiversity Corridor (NWC1: Protected Category VI) within an area housing a series of ELCs. The ELCs were established between 2005 and 2010, but only demonstrated an enhancement in deforestation from 2016 onward (2001–2015 average = 0.29%; 2016–2021 average = 1.56%). Similar trends were observed in NWC1 (2001–2015 average = 0.21%; 2016–2021 average = 1.30%) and less strikingly in PPWS (2001–2015 average = 0.10%; 2016–2021 average = 0.21%); with significant correlations between forest loss at the local ELCs and both NWC1 (R = 0.86, *p* < 0.0001) and PPWS (R = 0.87, *p* < 0.0001). A positive but insignificant correlation was found between the ELCs and KSRP, likely highlighting the minor increasing trend from 2016 onward in KSRP. While this deforestation rate is still very low in KSRP, it is worthwhile to note for management purposes.

#### Central block

The central protected area block region (block + 10 km buffer) has lost a total of 37.9% forest cover in the past two decades (2001–2021), representing a 29% higher rate than the country-level average. The average annual forest loss rate has more than tripled from 0.80 from 2001 to 2010 to 2.72% from 2011 to 2021. Within the central block (Fig. 2aii), the Tumring REDD+ Project (TRP; Fig. 1a; Supplementary Fig. 2) shares 85% of its borders with unprotected high deforestation land (and ELCs) and the remaining 15% with Prey Lang Wildlife Sanctuary (PLWS: Protected Category IV, established in 2016) in the NE. Rates of deforestation in TRP were compared to those in other category VI (two segments of the Northwest Biodiversity Corridor: NWC2, NWC3) as well as higher level protected category IV Wildlife Sanctuaries (Beng Per and Prey Lang). Significant deforestation first initiated in ELCs located to the south of TRP, peaking between 2010 and 2013 (average annual = 13.1%, 2001–2021_total_ = 83.8%), followed by ELCs established within Beng Per Wildlife Sanctuary (BPWS: 2011–2013 average annual = 14.5%, 2001–2021_total_ = 72.9%), NWC2 (2012–2016 average annual = 2.9%, 2001–2021_total_ = 39.5%) and unclassified areas bordering TRP (2011–2016 peak average annual = 9.6%, 2001–2021_total_ = 81.5%). The ELCs established within BPWS represented nearly 30% of the land, accounting for 47% of PA’s 2001–2020 deforestation. A significant positive correlation was found between deforestation in BPWS ELCs and the remaining BPWS (R = 0.72, *p* < 0.001).

As deforestation levels begin to decline in the aforementioned areas, a rise in deforestation is apparent in TRP (2016–2021 average annual = 5.4%, 2001-2021_total_ = 37.6%), PLWS (2016–2021 average annual = 1.9%, 2001–2021_total_ = 18.8%) and NWC3 (2016–2021 average annual = 2.6%, 2001–2021_total_ = 23.8%). Despite the protected status of the Category IV and Category VI areas in the region, they have lost an average of 31.7% of forest cover. The TRP was more effective than 2 of the 4 other surrounding protected areas. No relationship was apparent between the category level of protection and effectiveness of protection in the central block.

#### Southwestern block

The southwest protected area block region (block + 10 km buffer) has lost a total of 11.5% forest cover in the past two decades (2001–2021), representing a 61% lower rate than the country-level average. The average annual forest loss rate has nearly doubled from 0.39 from 2001 to 2010 to 0.69% from 2011 to 2021. The increase in deforestation has resulted in almost 30% total forest loss across all four protected areas, most of which occurred within Phnom Samkos (PSWS) and Phnom Aural (PAWS) Wildlife Sanctuaries (14.8% and 11.0% forest loss, respectively). The Southern Cardamom REDD + area deviated from the other protected areas, demonstrating a significant decline in the average annual deforestation rate (−50%) since the REDD+ project initiated (2015–2020) compared to previous years (2001–2014). Conversely, deforestation rates increased in the adjacent PSWS (+ 219%), Phnom Kravanh National Park (PKNP: + 200%) and AWS (+ 130%) protected areas along the same timeline.

PKNP and AWS are bordered to the east by a series of ELCs founded between 2000 and 2011, three of which were established within AWS borders between 2009 and 2011. Significant positive correlations were found between the forest loss in these local ELCs and forest loss in both Aural (R = 0.90, *p* < 0.0001) and less robustly in Kravanh (R = 0.67, *p* < 0.0001).

## Discussion

### Deforestation trajectories and economic drivers

Cambodia has undergone significant forest loss in recent decades—with 2.6 million hectares of forest cover loss occurring since 2001, equating to 29.5% of forest cover^7^ and 1.45 billion tonnes of CO_2_ emissions^8^. The deforestation rates have increased by 76% in the last decade (2011–2021) compared to the previous (2001–2010; Fig. 1b)^7^. We find forest loss has occurred within three distinct Phases demonstrated by changepoint analysis: (1) Phase 1: steady rise from 2000 to 2009 (average = 0.82%/year), (2) Phase 2: peak years from 2010 to 2013 (average = 2.3%/year), (3) Phase 3: moderate phase from 2014 to 2021 (average = 1.6%/year). Whilst the annual rate of deforestation has declined since the Phase 2, Cambodia currently has the highest country-level annual rate of forest loss globally^7^, illustrating the relentless deforestation spreading across the landscape. Critically, much of this forest loss and degradation is occurring in mature primary forests (Fig. 1b), which hold significant carbon and are home to rich biodiversity and keystone species^17–19^.

This deforestation in Cambodia has been attributed to the widespread development of Economic Land Concessions (ELCs), the expansion of numerous agricultural frontiers and relentless illegal logging^20–22^. These drivers have been abetted by the establishment of an extensive national road network (Fig. 1a)^20^—developed to promote economic growth and urban–rural connectivity^23^. The majority (88.4%) of these roads have been funded by foreign governments (the People’s Republic of China: 38.5%, Japan: 37.9%, and Republic of Korea: 12.0%)^18^—all of whom have established land concessions within Cambodia’s borders^24^ through the allocation of state land into private land for long-term industrial plantations^22,25^. The expansion of ELCs across Cambodia (average addition of 105,000 ha/year of ELC land since 1998) has been directly attributed to up to 40% of the country’s deforestation^21^, with further indirect impacts due to encroachment into rural community lands (indigenous areas, community forests, subsistence agricultural fields). This results in landlessness, poverty, and land conflicts, forcing communities to migrate in search of arable land, further contributing to the growing degradation and destruction of forests^22,26–29^.

#### Strategic government intervention

Protected areas expanded across Cambodia in 1993 following a royal decree^26^; the legal details of which were delineated in the 2008 Protected Areas Law, introducing protected categories, wildlife corridors and strict laws prohibiting development^9^. While over 80 protected areas currently exist covering 35% of Cambodian land^10^, they are still under substantial threat^30^. In further efforts to curb deforestation, the Royal Government of Cambodia ordered the suspension of new ELCs and revocation of a subset of existing ELCs in 2012 (Order 01BB)^31^. This resulted in a reduction of ELCs from a peak of ~ 2.1 million ha in 2012 to ~ 1.6 million ha from 2014 onward (Fig. 1b), with a significant positive correlation between the quantity of land classified as ELCs and the country-level deforestation rate (R = 0.87, *p* < 0.0001), hinting at the positive impact of Order 01BB on the trajectory of Cambodia’s deforestation rate.

This order was followed by a strategic plan to establish and safeguard a network of protected areas in 2016^30^ (Fig. 1b). However, forest concessions in Cambodia tend to be located adjacent to protected areas—the latter of which have limited physical boundaries (fences and signs), insufficient on-the-ground enforcement, as well as a significant lack of investment and active management—enabling land grabbing, laundering of timber through legitimate ELCs, as well as the illegal harvest and transport of wood by armed groups^17,30,31^. In all three protected area blocks, we find significant correlations between the deforestation in local ELCs and adjacent protected areas (R = 0.67–0.90, *p* < 0.0001)—providing evidence of illegal ELC-originating deforestation beyond concessionary boundaries, as reported by environmental groups^11^. ELCs bring roads and other infrastructure to new areas, making previously isolated forests more accessible for secondary deforestation. Whilst Graham et al.^5^ confirmed that protected areas are more effective at reducing forest loss than non-protected control areas, protected status alone does not solve the root of the problem: economic needs and aspirations.

#### REDD+ effectiveness

Within the three protected area blocks and buffer zones, all have demonstrated recent significant increases in the rate of annual deforestation (Supplementary Table 2). In two of the three cases, the REDD + projects (KSRP and SCRP) were 158% more effective at protecting against forest loss compared to the adjacent protected areas. Prior to REDD + status, these project areas had a history of international NGO presence (albeit with limited financial resources) and relatively lower background deforestation rates compared to the country average (Supplementary Tables 1, 2). Keo Seima has been classified as protected since 2002 (Category V: Conservation Landscape) with subsequent conservation status upgrades until it was officially named a Wildlife Sanctuary (Category IV) in 2016. On the other hand, despite historic NGO presence, Southern Cardamom was only named a National Park (Category II) in 2016. While these projects did not eliminate forest loss, they decreased the average rate of deforestation by 50% from the project onset onwards.

Following REDD + initiation, KSRP and SCRP invested significant resources from carbon revenue into the development of extensive patrolling and security programs backed by law enforcement rangers— including the hiring of local community members who are often left at the frontier of land conflicts^32,33^ in addition to government-backed rangers. This investment has resulted in robust enforcement activity: for example, > 32,000 patrols have been conducted in SCRP since the REDD + project initiated in 2015, resulting in the confiscation of 6940 chainsaws, removal of 231,061 snares, seizure of 3382 logs and the rescue of nearly 2800 live animals (Supplementary Table 3). Whilst Wildlife Alliance was present in the area since 2002, the REDD + project provided substantial financial support and capacity, resulting in an average of a threefold increase in all enforcement outcomes (Supplementary Fig. 3). Furthermore, additional carbon revenue was invested in developing an ecotourism venture at SCRP, which has welcomed nearly 20,000 guests since 2017, generating $767,000 in revenue for the 5685 participating community members (Supplementary Table 4). While ecotourism was initiated prior to the REDD + project, the additional support of carbon revenues resulted in a 2.3-fold increase in the annual income generated despite the impact of the global pandemic.

In the final case, the TRP was 12% more effective than 50% of the surrounding protected areas and 79% less effective than the remaining protected areas. No relationship between the protected category level and effectiveness was found in this region. TRP and the surrounding PAs (Category IV: Prey Lang Wildlife Sanctuary and Beng Per Wildlife Sanctuary; Category VI: Northwest Biodiversity Corridor) are located within a deforestation hotspot with a 54% higher deforestation rate (2011–2021) than the Cambodia average, resulting in a total of 37.9% forest cover loss in the block since 2001 (Cambodia average = 29.5%). TRP has been classified at a low level of protection (26% of the area is Category VI: Community Forest) with no historic NGO presence and very limited carbon revenue (REDD + credits issued: 2018, first credits sold: 2021). Given the lack of both financial resources and foundational protection activity in TRP—combined with the extreme deforestation risk—it is unsurprising that this REDD + project has been less successful than the other REDD + initiatives (SCRP, KSRP) in Cambodia thus far. Despite the limited funding, TRP has initiated community-based patrols; however, backing by law enforcement limited and patroling remains restricted in scale at present^34^. Even if TRP carbon credits are sold at a stable rate going forward, the project has far less credits per unit area compared to the other REDD + project in Cambodia (TRP: 2.4 tCO2e/year/ha, SCRP: 6.2 tCO2e/year/ha, KSRP: 11.9 tCO2e/year/ha; Supplementary Table 5) due to the lower carbon density of TRP forests. Furthermore, there is often a delay between project initiation and investment of carbon finance due to administrative bottlenecks—thus, it is too soon to predict how successful TRP will be in the long-term. However, TRP is lacking certain characteristics that are associated with SCRP and KSRP, including: historic NGO presence, stable funding from carbon revenues, high-level protection status and effective patrolling to secure the area.

REDD + projects have faced criticism in the past—including citations of limited protection success^35^, weak community involvement (through Free, Prior and Informed Consent)^36^, insufficient sharing of carbon revenues with the communities^37^, risks of community displacement^38^ and a lack of community-run governance structure^39^—and not all have been successful^35^. However, those REDD + projects currently in operation in Cambodia hold the CCB (Climate, Community & Biodiversity) standard, demonstrating a commitment to achieving, and documenting through independent verification audits, net positive impacts to the local community and wildlife. Despite the TRP commitment to strong community involvement^34^, based on the lack of historic presence in the area and limited carbon revenues available for community sharing, the relationship and trust building with the community may take more time than KSRP and SCRP. Nevertheless, given the high deforestation risk in the area and presence of ELCs, it appears unlikely that the level of community involvement is a major factor leading to a lower success rate thus far at TRP.

#### REDD+ prospects

The results presented highlight the significant deforestation encroaching upon PAs and REDD + project boundaries in Cambodia, with ELCs associated with PA deforestation. The Royal Government of Cambodia has taken important steps in mitigating the deforestation crisis in Cambodia through the halting of new ELCs^26^—resulting in a decrease in annual deforestation from 2014 to 2021 compared to the 2010–2013 peak.

The government also initiated a National REDD + Strategy in 2017^40^, converting underfunded PAs into REDD + projects with the goal of developing a nested national REDD + system. In theory, carbon revenues would provide the adequate resources to enact rigorous boots-on-the-ground enforcement^8^ unavailable in national protected areas, and to provide material incentives to local government and the agents of deforestation through which the value of the standing forest could be comparable to the opportunity cost of forest conversion. If successful at scale, this strategy could work to reduce threats of non-permanence and leakage by transforming economic relationships between the people, the government and the forest at a national level, through a durable market-oriented policy and regulatory framework.

Thus far, the Southern Cardamom and Keo Seima REDD + projects demonstrated significantly higher effectiveness in reducing deforestation compared to adjacent PAs, while the Tumring REDD + project was less effective than 50% of the surrounding PAs. We hypothesize that a series of key enabling conditions are required for REDD + success in Cambodia, including: a history of on-site NGO presence and/or long-term conservation activities, rigorous boots-on-the-ground protection backed by law enforcement, stable funding from carbon revenues and a high-level of national protection status. Two new REDD + projects are currently being initiated in Cambodia in collaboration with the government: Northern Plains Landscape (NPL, initially comprising three PAs: Supplementary Table 6) and Phnom Samkos Wildlife Sanctuary (PSWS). Both of these project locations hold the above-mentioned enabling characteristics (Supplementary Table 7) and therefore appear to have favorable conditions in place for successful implementation.

Based on our findings, when implemented in association with favorable enabling conditions, REDD + initiatives have strong potential to ensure more effective long-term protection than national protected areas alone within high deforestation landscapes, jurisdictions, and countries.

## Methods

Tree cover loss data from Hansen et al.^7^ was utilized through the Global Forest Watch (GFW: https://www.globalforestwatch.org/) platform to analyze the annual forest cover loss at country and regional levels; specifically, the analysis of three REDD + projects (Keo Seima REDD + Project, Tumring REDD + Project and Southern Cardamom REDD +) and a series of surrounding and adjacent national protected areas (Keo Seima Wildlife Sanctuary, Snuol Wildlife Sanctuary, Phnom Samkos Wildlife Sanctuary, Phnom Kravanh National Park and Aural Wildlife Sanctuary, Phnom Prech Wildlife Sanctuary, Northwest Biodiversity Corridor, Beng Per Wildlife Sanctuary, Prey Lang Wildlife Sanctuary) and Economic Land Concessions (ELCs). Within each block, the deforestation rate was analyzed with a 10 km radius to investigate the regional forest loss. The shapefiles of the REDD + projects were obtained from the developers and uploaded to the GFW dashboard for analysis. The areas for the adjacent national protected areas were obtained from a GFW map layer from Open Development Cambodia: Natural protected areas in Cambodia (1993–2021)^30^. The areas for Economic Land Concessions (ELCs) were obtained using a dataset by LICADHO Cambodia^25^. The annual forest loss (ha, % area) was recorded for each year from 2001 to 2021 with a spatial resolution of 30 m. This data was used to calculate the average forest loss and annual deforestation rates at different time intervals.

Correlation analysis of deforestation rates in various land use types was completed using Pearson correlation, performed in R with the “ggpubr” package. Changepoint detection was performed on Cambodia forest loss data^7^ using the R package “changepoint” by identifying significant changes in the mean from 2001 to 2021.

## Supplementary Information

Supplementary Information.

## Data Availability

The datasets used and/or analyzed during the current study are available on the OSF database. Pauly, M. (2022, February 10). Voluntary REDD+ project areas avoid more forest loss than national protected areas in Cambodia. Retrieved from osf.io/xq2bz https://doi.org/10.17605/OSF.IO/XQ2BZ.
