# Supplementary material for: High deforestation trajectories in Cambodia slowly transformed through economic land concession restrictions and strategic execution of REDD+ protected areas
Source: Sci Rep. 2022 Oct 14;12:17102. doi: 10.1038/s41598-022-19660-0 (PMC9568659; doi:10.1038/s41598-022-19660-0)
Supplement: Supplementary file 1 — Supplementary Information. [file 41598_2022_19660_MOESM1_ESM.pdf]

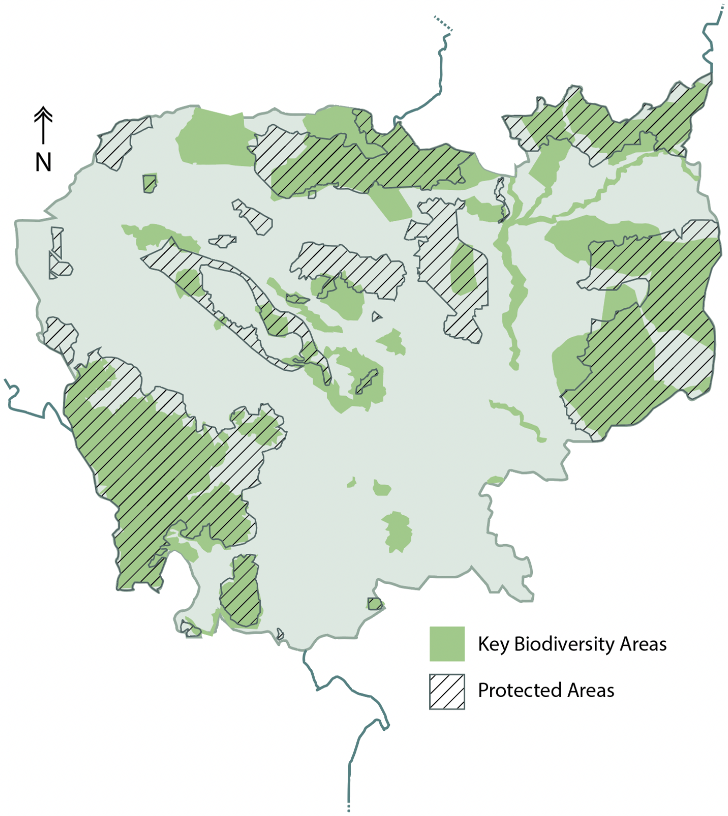

Supplementary Figure 1. Protected Areas[6] and Key Biodiversity Areas[41] in Cambodia.

REDD+ Project	REDD+ start year	Protected Status year	Protected Status	High threat borders	NGO Presence	Zone deforestation (since 2001)
Keo Seima Wildlife Sanctuary	2010	2002, 2016	Category IV (Wildlife Sanctuary): core zone of high conservation value, access prohibited	64%	Wildlife Conservation Society present since 2002	15.4%
Tumring	2015	2008-2010	Category VI (Community Forest): sustainable use of natural resources	85%	No NGO presence	37.9%
Southern Cardamom	2015	2016	Category II (National Park): high conservation value with access permitted for recreation and tourism	47%	Wildlife Alliance present since 2002	11.5%

Supplementary Table 1. A summary of the REDD+ projects registered in Cambodia. IUCN Management Category Protected status refers to the year the region received official protection category status from the Royal Government of Cambodia. High threat borders estimated based on the percentage of REDD+ project borders adjacent to unprotected land. Zone deforestation is defined as the total cumulative forest loss as a percentage of the area within the protected area blocks (Figure 2) with a 10km buffer.

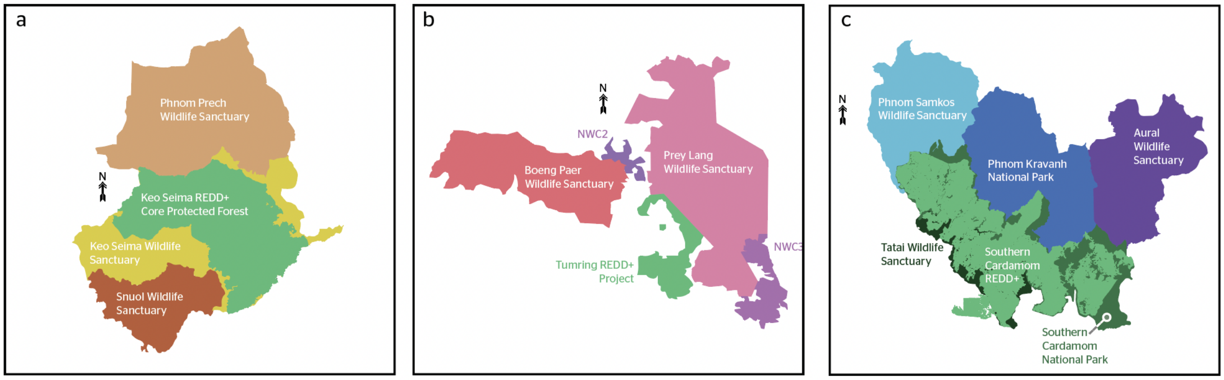

Supplementary Figure 2. The three protection area blocks analyzed: (a) Eastern Block, (b) Central Block and (c) Southwestern Block. Protected area layers accessed on Global Forest Watch[7], mapped on ArcGIS v10.9, finalized on Adobe Illustrator v26.2.1[41].

**Deforestation**	**Block 1: Eastern**	**Block 2: Central**	**Block 3: Southwestern**	**Cambodia (country-level)**
2001-2010 (annual average)	0.45%	0.80%	0.39%	1.01%
2011-2021 (annual average)	0.99%	2.72%	0.69%	1.76%
2001-2021 (total)	15.4%	37.9%	11.5%	29.5%

Supplementary Table 2. A summary of the average annual deforestation rates and total deforestation occurring within the studied 3 protected area blocks compared to the country-level.

Enforcement Indicator	Pre-REDD+ (2002-2015)		Post-REDD+ (2016-2022)		REDD+ improvement
	Total	Average annual	Total	Average annual	
# of patrols	22,543	1,734	32,220	3,580	+106%
# of signs installed	10,716	824	7,696	855	+4%
# logs seized	37,723	2,902	30,438	3,382	+17%
# chainsaws confiscated	1,011	78	6,940	771	+892%
# snares removed	113,898	8,761	231,066	25,674	+193%
# live animals rescued	4,234	423	3,798	422	0.1%

Supplementary Table 3. A summary of enforcement data for the Southern Cardamom REDD+ project, provided by Wildlife Alliance (2022). The pre-REDD+ period refers to the time when Wildlife Alliance was present on the ground conducting protection activities prior to the initiation of the REDD+ project. The post-REDD+ period refers to the point of the Southern Cardamom REDD+ project onward, during which Wildlife Alliance (in collaboration with the Royal Government of Cambodia) stepped up enforcement activities through the utilization of carbon finance from the REDD+ VCS sales.

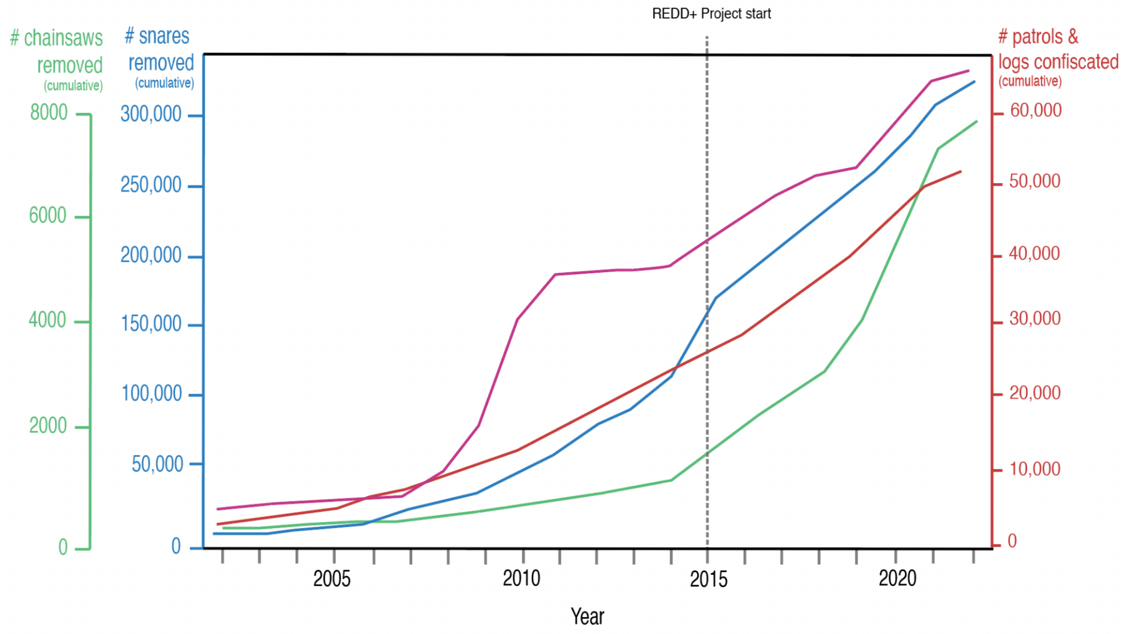

Supplementary Figure 3. Boots-on-the-ground enforcement results from the Southern Cardamom area, data provided by Wildlife Alliance (2022). Pre-2015 results demonstrate activities prior to REDD+ initiation. Data summarized in Supplementary Table 3.

	# of tourists	Income generated (USD)
Pre-REDD+ total (from 2008)	18,505	$608,116
Pre-REDD+ annual average	2056	$67,568
REDD+ total (from 2017)	19,307	$766,932
REDD+ annual average	3861	$153,386

Supplementary Table 4. Eco-tourism data from the Southern Cardamom REDD+ project, data provided by Wildlife Alliance (2022).

**Project**	**tCO2e/year achieved**	**Project area**	**tCO2e/year/ha**
Southern Cardamom REDD+ Project	5,521,098	27,605,489	11.9
Keo Seima REDD+ Project	1,819,837	16,378,530	6.2
Tumring REDD+ Project	161,353	645,410	2.4

Supplementary Table 5. A summary of the VCU (verified carbon units) in tCO2e, averaged annually based on the verified VCUs achieved by the project to date. The data tCO2e/year and project area were obtained from project verification reports and project description documents, respectively, publicly on the Verra Carbon Standard Registry accessible at: https://registry.verra.org/app/search/VCS.

**Protected Area**	**Protected Status Details**
Chhep Wildlife Sanctuary (CWS)	Designated in 2002 as a Protected Forest, upgraded to a Wildlife Sanctuary in 2016
Preah Roka Wildlife Sanctuary (PPRWS)	Designated as a Wildlife Sanctuary in 2016
Kulen Promtep Wildlife Sanctuary (KPWS)	Designated as a Wildlife Sanctuary in 1993
Phnom Tbeng Natural Heritage Park (PTNHP)	Designated as a Heritage Park in 2016

Supplementary Table 6. A summary of Protected Areas making up the upcoming Northern Plains Landscape REDD+ project, developed by the Wildlife Conservation Society (WCS) in collaboration with the Royal Government of Cambodia.

Characteristic	KSRP	SCRP	TRP	NPL	PSWS
Low - moderate deforestation rates (at or below the country-level average)	✓	✓	✖	✓	✓
Historic NGO presence	✓	✓	✖	✓	✓
Protection activities backed by law enforcement	✓	✓	✖	?	?
Stable carbon credit funding	✓	✓	✖	?	?
REDD+ status backed by high-level national protection category	✓	✓	✖	✓	✓

Supplementary Table 7. Characteristics of REDD+ projects in Cambodia, categorized for current (KSRP: Keo Seima REDD+ Project, SCRP: Southern Cardamom REDD+ Project, TRP: Tumring REDD+ Project) and future (NPL: Northern Plains Landscape REDD+ Project, PSWS: Phnom Samkos Wildlife Sanctuary REDD+ Project) according to the presence (✓), absence (✖) and not confirmed (?) status.
